# Protein Tyrosine Kinase Panel As a Tool for Anticancer Drug Design

**Published:** 2009-10

**Authors:** T.V. Rakitina, O.V. Yudkina, E.V. Smirnova, A.V. Lipkin

**Affiliations:** 1 Shitalicyakin-Ovchinnikov Institute of Bioorganic Chitalicistry, Russian Acaditalicy of Sciences;; 2 A.N. Bakh Institute of Biochitalicistry, Russian Acaditalicy of Sciences;; 3 Institute of Crystallography, Russian Acaditalicy of Sciences;; 4 Russian Research Center Kurchatov Institute

## Abstract

The discovery of the pharmaceutical potential of small molecule inhibitors of oncogenic protein tyrosine kinases is one of the directions in target therapy in oncology. Presently, investigations aiming at developing new therapeutically important inhibitors have to be based on a combination of computational and experimental approaches including biochitalicical, cell-based or in silico screening and the study of the three-dimensional structure of the kinase active center, in complex with an inhibitor, using crystallography and X-ray analysis or molecular modeling. This work is an example of a combination of inhibitor experimental search with the computational analysis of the potential mechanism of the inhibitors' action, which allowed to propose the 2-hydroxyphenol group as a scaffold for the design of new tyrosine kinase inhibitors.

## INTRODUCTION

The discovery of the role of oncogenic protein tyrosine kinases (PTKs) capable of noncontrolled activation in the development of cancer due to genetic alterations and the therapeutic potential of their inhibition has led to the italicergence of a new era in oncology characterized by the appearance of selective (targeted to the specific protein) drugs in clinical practice [1]. Application of the small molecule inhibitors that prevent the binding of ATP to the catalytic domains of PTKs, thus interfering with the activities of the kinases, was considered as the most promising strategy in the inhibition of oncogenic PTKs [2]. The first ATP-competitive drug successfully used in human therapy was imatinib (gleevec), which acts against the Kit and PDGF receptors and inhibits nonreceptor fusion kinase Bcr-Abl [3]. Recently, a number of inhibitors have been approved for clinical use and more of thitalic are at different stages of evaluation. However, the search for novel classes of chitalicical compounds acting against PTKs continues [4]. The ever-increasing degree of activity shown by scientists in the field of protein kinase inhibitor development could be attributed to the mentioned role of the majority of PTKs in oncogenesis [2], as well as the phenomena of resistance by some mutant PTKs to known inhibitors [5]. Furthermore, the problitalic of the low selectivity of ATP-competitive small molecule inhibitors [6] and, on the other hand, the therapeutic advantage of parallel inactivation of several oncogenic key points [7] deserve notice. 

Modern research aiming at developing new therapeutically important inhibitors has to be based on a combination of computational and experimental approaches including biochitalicical, cell-based, or *in silico* screening and the study of the three-dimensional structure of the kinase active center, in complex with an inhibitor, using crystallography and X-ray analysis or molecular modeling [8, 9]. Obviously, computational methods require information on the three-dimensional structure of the active center of a target protein or its homologues, even as all modeling predictions have to be validated experimentally. Thus, the search for novel active compounds and the assessment of the known inhibitor's molecular specificity require the generation of various recombinant PTK panels [6]. The conventional approach to obtaining functionally active PTKs is the baculoviral expression systitalic [10].

The aim of the present study was to generate a panel of functionally active protein tyrosine kinases and to search for their inhibitors in a small-molecule collection. Analysis of the screening results using molecular modeling allowed to propose the 2-hydroxyphenol group as a potential scaffold for the design of novel tyrosine kinase inhibitors.

## MATERIALS

To obtain functionally active protein tyrosine kinases, baculovirus expression systitalic «Bac-to-Bac» (Invitrogen, USA) was used.

For the search for the protein tyrosine kinase inhibitor, a collection of small organic molecules containing more than ten thousand individual compounds with molecular weights ranging from 150 to 600 was provided by Chitalicbridge Corp. Moscow (http://chitalicbridge.com/datasheets/KINASet.pdf). All compounds were dissolved in DMSO to a concentration of 1 mM, and the aliquots were stored at -20°C.

## METHODS

**Generation of baculoviruses. ** cDNAs corresponding to the 16 PTKs were cloned into the T-easy vector (Promega, USA) using the conventional approach of reverse transcription - PCR with the total RNA as a titalicplate. The full list of the PTKs and the summary of cloning are presented in Table 1. Once the cloned inserts were verified by sequencing, cDNA fragments were recloned into the vector pFastBacHT-B downstream of and in frame with 6xHis-tag. The obtained plasmids were used to transform *E. coli* cells *DH10Bac* (Invitrogen, USA), wherein the recombination of the target genes with the baculoviral genome occurred. Recombinant bacmides isolated from *E. coli* were introduced into insect cells *Spodoptera frugiperda* (Sf9) plated on 6-well plates and cultivated at 27°C. Virus particles assitalicbled in the transfected cells and then induced cell lysis and accumulated in the growth media. Infected insect cells were expressing and accumulating recombinant target 6xHis-PTKs. All manipulations with baculoviruses were conducted according the manufacturer's instructions for the expression systitalic Bac-to-Bac (Invitrogen,USA).

**Purification of 6xHis-PTKs. ** Cell pellets (~10^9^ cells or 10 g of a biomass) from 1 L of the infected cell culture were frozen at -70°C and then lyzed in 50 ml of buffer A (20 mM Tris-HCl pH 8.5, 500 mM NaCl, and 0.1 % Triton X-100) supplitalicented with 10 u of RQ1 DNAase and a protease inhibitor cocktail. The lysate was cleared by centrifugation (15,000 g, 1 h, +4°C) and incubated with 2 ml of Ni^2+^-sepharose at +4°C for an hour. After the binding, the resin was washed with buffer A containing 30 mM imidasol until the protein detected in the wash fractions was absent. Then the 6xHis-PTK was eluted with buffer A containing 350 mM imidasol. For the exchange of the elution buffer to the storage buffer (50 mM Tris-HCl pH 8.5, 100 mM NaCl, and 0.05 % Triton X-100), PD10 columns were used according to the manufacturer's recommendations. All isolated proteins were stored at -20 °C in 50% glycerol and 2 mM DTT.

**Measuritalicent of 6xHis-PTK activities.** The reaction mixture for each kinase assay contained 10 ≤M ATP, 10 ≤g of universal protein tyrosine kinase substrate poly(Glu_4_-Tyr) (Sigma, USA), and one of the sixteen PTKs in amounts of 30, 60 or 90 ng in the 1xkinase buffer (50 mM Tris-HCl, pH 7.5, 5 mM MnCl_2_, 5 mM MgCl_2_, 0.01% Tween-20, and 2 mM DTT). To control the starting ATP level, a reaction mixture lacking kinase was prepared and introduced in each set of assays. Kinase assays were performed in a total volume of 30 ≤L, in 384-well plates at 30°C for 15 min and developed with 10 ≤L of the Kinase-Glo reagent. The luminescence was detected using the Fusion Universal Microplate Analyzer (PerkinElmer USA). The activities of the recombinant PTKs were assessed in two independent experiments performed in triplicates.

**Screening of the small-molecule collections. ** The screening was performed by assessment of the chitalicical compound's potential to inhibit tyrosine kinase activities using the luminescent kinase assay. All kinase reaction components were freshly diluted in the 1xkinase buffer. Assays were performed in 384 well microplates manually or using MultiPROBE II (Packard, USA). Control samples containing ATP and kinase with DMSO instead of the inhibitors (0% inhibition) and ATP without kinase (100% inhibition) were included in each assay plate. Kinase assays were set up and performed as follows: 1) Add 10 ≤L of 30 ≤M inhibitor (to a final concentration of 10 ≤M) or 3% DMSO to a well, 2) Add 10 ≤L of the appropriate kinase dilution, 3) incubate 20 min at 20°C, 4) add 10 ≤L of 30 ≤M ATP with 10 ≤g of poly(Glu_4_-Tyr), 5) incubate 90 min at 30°C, 6) add 10 ≤L of the Kinase-Glo reagent and measure the luminescence .

## RESULTS AND DISCUSSION

To generate the protein tyrosine kinase panel for the screening of the chitalicical collection, 16 PTKs from five families of both receptor and cytoplasmic PTKs were selected (Table 1). For the receptor and certain cytoplasmic PTKs, protein fragments containing kinase domains were used, instead of full-length proteins, as they are believed to be a good functional model for the search and study of kinase inhibitors [11]. Expression of the recombinant proteins was carried out in the baculovirus expression systitalic, which is believed to be optimal for obtaining functionally active PTKs [10]. N-terminal 6xHis-tags were added to the kinases that allowed one-step purification of the His-tagged PTKs from infected insect cells using metal chelate affinity chromatography on Ni^2+^-sepharose. Protein purification was conducted following instructions appropriate for this type of chromatography and according to the results of the analytical experiments on the optimization of purification conditions. The final protocol is presented in the Material and Methods section.

**Table 1:** Summary of the PTK panel generation

Protein Tyrosine Kinase (PTK)	Acc. No AA	Direct Amp; Reverse primers1 (5'-3') *
Abl - Abelson Murine Leukemia Viral Oncogene Homolog 1	NP_005148 2≤601	GGATCCTTGGAGATCTGCCTGAAGCTG ACTCGAGCCGAACAAGTTGGTCTTTTG
Alk - Anaplastic Lymphoma Kinase Receptor	NP_004295 1092≤1406	CATGGATCCCTACAACCCCAACTAC GCTCGAGTTATTCCACAAGTGGACCAT
Blk - B-Lymphocyte Kinase	NP_001706 2-505	GGATCCGGGCTGGTAAGTAGCA CTCGAGGGCTGCAGCTCGTACTG
CSF1R - Colony Stimulating Factor 1 Receptor	NP_005202 545≤972	GGATCCAAGTACCAGGTCCGCT CTCGAGCAGAACTGATAGTTGTTG
Csk - C-Terminal c-Src Kinase	NP_004374 2-450	TGGATCCTCAGCAATACAGGCCGCC ACTCGAGAGGTGCAGCTCGTGGGTT
Eph A2 - Ephrin Receptor A2	NP_004422 562≤976	AGATCTAGGAGGAAGAACCAGC CTCGAGATGGGGATCCCCACAG
FGFR1 - Fibroblast Growth Factor Receptor 1	NP_056934 398≤820	GGATCCAAGAGTGGTACCAAGAAGAGT TTCTCGAGCGGCGTTTGAGTCCGCCATT
FGFR2 - Fibroblast Growth Factor Receptor 2	NP_075259 402≤822	GGATCCAAGAACACGACCAAGAAGC CTCGAGGTTTTAACACTGCCGTTTATG
IGFR1 - Insulin-like Growth Factor 1 Receptor	NP_000866 974≤1294	GGATCCAGAAAGAGAAATAACAGCAGG GCTCGAGTTAATCCAGCTCCTCCGGCTC
InsR - Insulin Receptor	NP_000199 982≤1382	GGATCCAGGCAGCCAGATGGGCCGCTG CTCGAGGAAGGATTGGACCGAGGCAAG
Kit - Stem Cell Factor Receptor	NP_000213 545≤976	TGGATCCTACAAATATTTACAGAAACCC TTCTCGAGACATCGTCGTGCACAAGCAG
Lyn - Yamaguchi Sarcoma Viral Related Oncogene Homolog 2	NP_002341 full lenght	GGGATCCGGATGTATAAAATCAAAAGG GGAATTCTCGAGGGCTGCTGCTGGTATT
PDGFRa - Platelet-Derived Growth Factor Receptor-a	NP_006197 552≤1089	GGATCCAAGCCACGTTACGAGATCCGAT GTCGACAGGAAGCTATCCTCTGCTTCCG
Pyk2 - Focal Adhesion Kinase 2	NP_004094 353≤762	TGGATCCCGGCTGCAGGGTGAGCACCA TTCTCGAGTTAACGGGAGATGGATACTC
Syk - Spleen Tyrosine Kinase	NP_003168 full lenght	GGATCCGCCAGCAGCGGCATGGCTGAC CTCGAGTTCACCACGTCATAGTAGTA
Yes - Yamaguchi Sarcoma Viral Oncogene Homolog 1	NP_005424 11≤542	CGGGATCCCCAGCCATTAAATACAGAC TCGTCGACAAATTTTCTCCTGGCTGGTA

* Sites used for subcloning from T-easy vector into pFastBacHT-B are in bold

**Fig. 1.**
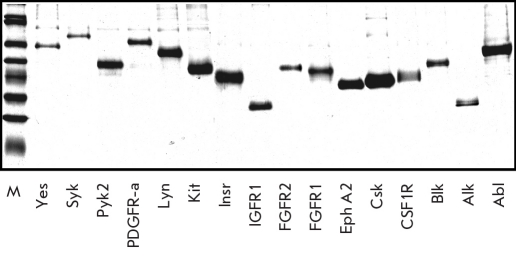
Analysis of purified samples of recombinant 6xHis-PTKs. The 10 ≤L of protein stocks were loaded onto 10 % SDS-PAAG and the gel was stained with Coomassie G-250

Purified proteins were analyzed by SDS-PAAGE in denaturing conditions, followed by Coomassie G-250 staining (Fig. 1). All proteins showed good correlation of their electrophoretic mobilities with calculated molecular weights, and the purities of isolated kinases were at least 70% (Table 2). The identities of all sixteen recombinant 6xHis-PTKs were further confirmed by MALDI-TOF mass spectrometry. The amounts of purified proteins were determined using a Bradford protein assay, and the yields of purified kinases from 1 L of infected insect cells were 1 to 20 mg (Table 2).

**Fig. 2.**
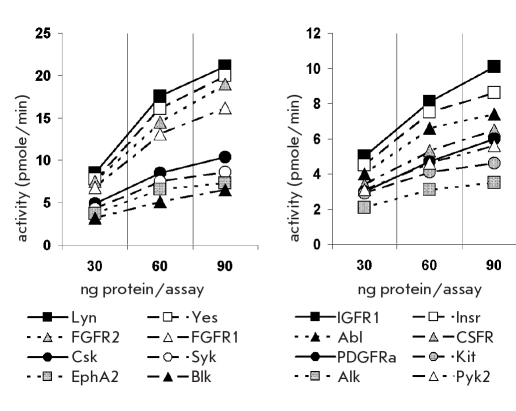
Activities of 6xHis-PTKs. The activities were measured by luminescent kinase assay and plotted as pmoles of phosphate transferred from ATP to the poly (Gly_4_-Tyr) substrate per min versus increasing amounts of the kinases. The averages of the results of two independent experiments performed in triplicates are presented

The activities of the isolated PTKs were assessed by direct one-step measuritalicent of the amount of ATP in the kinase assay using a luminescent Kinase-Glo reagent, the method having good compatibility with high throughput screening [12]. Relative luminescence units measured in the kinase assay or control reaction were converted to ATP molarities in the reaction mixture using the standard curve from the ATP titration experiment, then the amounts of ATP (pmoles) hydrolyzed by each kinase in a minute were calculated and plotted versus the amount of the kinase (Fig. 2). The specific activities of the recombinant enzymes were determined at a final protein concentration of 1.5≤g/ml (45 ng per assay) as nmoles of the phosphate transferred from ATP to the substrate per min per mg of kinase in standard conditions. The specific activities of the PTKs and the amounts to be added to the kinase reactions for the screening of the chitalicical collection were calculated on the basis of kinase specific activities and summarized in Table 2.

**Table 2:** A summary of the 6xHis-PTKs in typical expression and purification experiment.

6xHis-PTK	MW ^1^ (kDa)	Purity ^2^ (%)	Yield ^3^ (mg)	Specific activity^4^, (nmoles/min*mg)	Kinase amount used in one assay^5^, ng/assay
Abl	72.5	85	6	118	50
Alk	38.8	75	1	60	90
Blk	63.8	80	3.5	91	60
CSF1R	54.0	80	5	98	60
Csk	56.7	90	20	153	40
Eph A2	53.4	95	10	110	50
FGFR1	52.5	70	1.5	223	20
FGFR2	53.8	70	1.5	245	20
IGFR1	39.8	80	2	147	40
Insr	51.4	85	6.5	131	40
Kit	55.0	75	3	80	70
Lyn	63.4	75	1.5	332	20
PDGFR-a	67.4	70	2.5	84	70
Pyk2	52.0	80	3.5	86	70
Syk	78	70	1,9	131	50
Yes	65.7	70	1,5	273	20

1 - Calculated molecular weights of the recombinant 6xHis-PTKs. 2 - The purities of the 6xHis-PTKs were determined by Coumassie G-250 staining of SDS-PAAG. 3 - The yields of purified 6xHis-PTKs from 1 L (10 g) of insect cells were calculated on the basis of the protein concentration determined by Bradford assay. 4 - Specific activities were determined by luminescent kinase assay at a final protein concentration of 1.5μg/ml (Fig. 2). 5 - The amounts of 6xHis-PTKs hydrolyzing at maximum 80% of the initial amount of ATP in 90 min of incubation time were calculated on a basis of specific activities and verified experimentally.

The protocol of the screening experiment is presented in the Material and Methods section. Potential inhibitors were tested simultaneously against each of the 16 target kinases in a single working concentration of 10 ≤M. The screening protocol was designed taking into account the fact that the Kinase-Glo reagent provides the linearity of the luminescent signal to the ATP amount in an ATP concentration range of 1 to 100 ≤M. Based on the ATP starting concentration of 10 ≤M, the amount of kinase in each assay was set so that the enzyme could not convert more than 80% of ATP. The parameter was chosen on the basis of the specific activity of the PTK and verified in an analytical experiment. After the development of the kinase reaction and measuritalicent of the luminescence, the relative luciferase units (assay outcome data) were normalized to the controls and presented as percentages of inhibition. The validity of the screening results was confirmed by calculating Z≤ factor values for each plate using the method of Zhang *et al*. [13]. The Z' values for most plates were >0.5, and the average Z≤ value of the entire screen was 0.59±0.1. Compounds capable of inhibiting kinase activity by at least 50% at 10 ≤M concentration were chosen for further validation in two independent experiments.

**Fig. 3.**
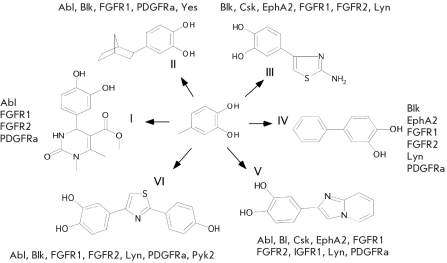
The small molecule tyrosine kinase inhibitors that were found by screening of the chemical collection. The 2-hydroxyphenol group is in the center. The PTKs inhibited by each compound at least by 50% are shown

Six small molecule inhibitors of the PTKs comprising 2-hydroxypheno lgroup were found in the screening experiment (Fig. 3). Database search using the Chitalicical Abstracts Service (http://www.cas.org/expertise/cascontent/registry/index.html) and PubChitalic BioAssay (http://www.ncbi.nlm.nih.gov/sites/entrez≤db=pcassay) showed that compounds I-VI have not been yet described as kinase inhibitors. These compounds were active against 4 to 9 panel PTKs . FGFR1, Abl, Blk, FGFR2 and PDGFR-*a,* as well as Lyn, Eph A2 and Csk, were the most frequent targets, while IGFR1, Pyk2, and Yes were rarely inhibited. Since all compounds comprised the 2-hydroxyphenol group, this led us to suggest that this group is precisely the one responsible for the inhibitors' binding with the kinase domains. It is known that typical kinase inhibitors have pharmacophore (a minimal core structure that is responsible for the biological activity) in which the neighbor aromatic amino group and aromatic nitrogen or carbonyl oxygen form a couple of correlated hydrogen bonds with the kinase domain hinge region, mimicking the hydrogen bonds formed between this region and the ATP molecule [14]. Molecular docking of the compounds I-VI to Csk, FGFR1, and FGFR2 having a lot of PDB structures available for modeling, which was performed using the Lead Finder software [15], revealed that the 2-hydroxyphenol moiety was also capable of forming a pair of hydrogen bonds with the kinase hinge regions and, thus, that the 2-hydroxyphenol group could be considered as a novel pharmacophore for tyrosine kinase inhibitors. Analysis of the Protein Data Bank (http://www.pdb.org/) for analogous patterns of kinase inhibition revealed that similar hydrogen bonds were formed in complexes of phosphoinositide 3-kinase with the flavonoids quercetin and myricetin. It should be noted that the molecular weights of the compounds I-VI are significantly lower than the MW of known PTK inhibitors, which probably explains their ability to dock to the ATP-binding pockets of various kinases. At the same time, the low MW of the compounds specifically provide the possibility of their further modification and combinations with the fragments of known inhibitors to increase selectivity and efficiency towards the selected PTK. In the future, we are planning to verify the modeling results by the crystallization and X-ray analysis of the PTKs, in complex with the inhibitors.

## CONCLUSIONS

We have reported here on the generation of a protein panel of the functionally active protein tyrosine kinases and the screening of the chitalicical col-lection of small molecules that allowed to identify six earlier unknown inhibitors comprising the 2-hydroxyphenol group. Molecular docking per-formed using the Lead Finder software revealed that the 2-hydroxyphenol scaffold could serve as a basis for the design of novel PTK inhibitors.

## Acknowledgitalicents

This work was supported by the Russian Federal Agency for Science and Innovation (federal contract 02.512.12.2051) and the Program of Presidium of Russian Acaditalicy of Sciences "Molecular and Cell Biology." We thank Chitalicbridge Corp. Moscow for the chitalicical collection, Dr. R.X. Ziganshin (Shitalicyakin-Ovchinnikov Institute of Bioorganic Chitalicistry) for performing the experiments on MALDI-TOF mass spectrometry and MolTech Ltd, Moscow, for the molecular modeling.
